# Photobiomodulation and sleep quality: systematic review and meta-analysis

**DOI:** 10.1007/s10103-026-04913-5

**Published:** 2026-06-20

**Authors:** Wellington Marcos Vital de Azevedo, Kevlin de Souza, Maria Luiza Defante, Afonso Shiguemi Inoue Salgado, Rodrigo Álvaro Brandão Lopes-Martins, Sérgio Gomes da Silva, Francisco Gonzalez-Lima, Fabrizio dos Santos Cardoso

**Affiliations:** 1 Hospital São Vicente de Paulo (HSVP), Bom Jesus do Itabapoana, Brazil; 2 Natural Quanta Wellness Center, Orlando, USA; 3 Universidade Brasil, São Paulo, Brazil; 4 Hospital do Câncer de Muriaé, Fundação Cristiano Varella (FCV), Muriaé, Brazil; 5 The University of Texas at Austin, Austin, USA

**Keywords:** Photobiomodulation, Low-level laser therapy, Sleep quality, Mitochondrial metabolism, Therapy for sleep disturbances

## Abstract

Sleep disturbances are increasingly prevalent and associated with altered brain metabolism and neuroinflammation. Photobiomodulation, a non-invasive technique using red or near-infrared light, can modulate mitochondrial activity, cerebral perfusion, and glial function, suggesting potential benefits for sleep regulation. This systematic review and meta-analysis evaluated the effects of photobiomodulation on sleep quality in clinical and experimental populations. A comprehensive search was performed in PubMed, Embase, and Cochrane databases through September 2025 using the terms (photobiomodulation OR “low level laser therapy”) AND sleep. Five randomized controlled trials involving 240 participants met inclusion criteria. The pooled analysis suggested that photobiomodulation improved the Pittsburgh Sleep Quality Index (PSQI) scores compared with sham-control (MD = − 1.25; 95% CI − 2.38 to − 0.11; *p* = 0.03, I^2^ = 36.2%), although the wide confidence intervals indicate limited precision of the estimate. Mechanistic and neuroimaging evidence supports that PBM enhances mitochondrial metabolism, modulates astrocytic activity, and reorganizes prefrontal networks involved in sleep regulation. Despite methodological heterogeneity among studies, these findings suggest that photobiomodulation may have potential as a non-pharmacological approach for improving sleep quality; however, the current evidence remains limited and should be interpreted with caution. Further large-scale, standardized, and multimodal trials are required to establish optimal stimulation parameters and confirm long-term efficacy and safety.

**Supplementary Information:**

The online version contains supplementary material available at 10.1007/s10103-026-04913-5.

## Introduction

Sleep is a fundamental physiological state, regulated by complex neurobiological mechanisms, and essential for maintaining health and well-being [33, 38]. During sleep, the body enters a phase of recovery and reorganization, with a reduction in the perception of external stimuli and motor activity, allowing the restoration of critical functions, such as energy replenishment and tissue repair [38]. Sleep quality can be influenced by various factors, including dietary habits [48, 52], genetic predispositions [16], and environmental conditions [2]. Additionally, sleep contributes to immune system strengthening, hormonal balance, and memory consolidation, making it a key factor in cognitive and emotional functioning [31, 33, 44].

Sleep disorders, such as insomnia, are highly prevalent and affect approximately one in four adults worldwide, according to the World Health Organization [41]. These disorders can have serious consequences, impairing both physical and cognitive performance, while also negatively impacting work performance, social interactions, and quality of life [19, 46]. These symptoms can often be debilitating and are associated with significant adverse outcomes for physical health and general well-being [14, 37, 58].

Medication use in clinical practice should be carefully assessed, as many drugs can cause adverse effects on various organs. For example, drugs like anti-inflammatory medications and those used to treat insomnia have been linked to multiple side effects [20, 26]. Moreover, prolonged use of opioids can lead to dependence and hinder recovery [63, 66], while hypnotic agents may cause tolerance, dependence, and rebound insomnia [36]. Therefore, non-pharmacological treatments may offer safer and longer-lasting alternatives. Among non-pharmacological approaches, cognitive behavioral therapy for insomnia (CBT-I) is considered a first-line treatment for chronic insomnia due to its effectiveness in improving sleep quality and sleep-related outcomes. Randomized clinical trials have demonstrated that CBT-I can significantly reduce insomnia severity, improve sleep efficiency, and produce sustained long-term benefits in patients with chronic insomnia [22, 34]. However, despite its clinical efficacy, CBT-I may present limitations related to accessibility, treatment adherence, availability of trained professionals, and variable response rates among patients [13, 21]. In this context, the investigation of novel non-invasive neuromodulatory interventions such as photobiomodulation may provide complementary or alternative strategies for individuals with persistent sleep disturbances or limited response to conventional therapies.

Photobiomodulation is a non-invasive technique that uses light at specific wavelengths (most commonly red-to-infrared) to interact with biological tissues and stimulate cellular respiration, tissue repair and neurochemistry modulation [8, 10, 45, 25]. In the central nervous system, photobiomodulation has been associated with enhanced mitochondrial activity, modulation of oxidative stress, nitric oxide release, and regulation of neuroinflammatory pathways [9, 10, 23, 61, 62, 64]. These mechanisms may directly influence sleep physiology and circadian regulation and may also contribute to previously reported cognitive and emotional effects of photobiomodulation [23, 57, 62, 64]. Experimental evidence also suggests that photobiomodulation may affect astrocytic metabolism and adenosine signaling, which are closely related to sleep homeostasis and sleep–wake regulation [54, 56]. In addition, photobiomodulation may influence cortical and thalamocortical networks associated with arousal regulation and sleep quality [32, 35]. Therefore, photobiomodulation has emerged as a potential non-pharmacological strategy for improving sleep disturbances.

Based on the well-documented effects of photobiomodulation on different brain functions and disorders [3–7, 18, 25, 27, 49, 51, 57, 65], this systematic review and meta-analysis aims to critically evaluate the available evidence on the effects of photobiomodulation on sleep.

## Methods

This systematic review and meta-analysis was conducted in accordance with the Cochrane Collaboration recommendations and the Preferred Reporting Items for Systematic Reviews and Meta-Analyses (PRISMA) guidelines [30, 42]. The study protocol was registered in the PROSPERO database in March 2025 under the ID CRD420251058722, where all analytical procedures and statistical methods were pre-specified.

### Eligibility criteria

Studies were eligible for inclusion if they: (1) enrolled participants at risk of impaired sleep quality or a diagnosed sleep disorder; (2) were randomized controlled trials (RCTs) evaluating photobiomodulation therapy; (3) employed a sham-control or placebo comparison group; and (4) reported sleep quality outcomes assessed with validated instruments. Exclusion criteria were: (1) studies without a control group; (2) studies not reporting relevant sleep outcomes; (3) non-randomized or observational designs; and (4) conference abstracts, study protocols, or reports without full data.

### Search strategy and data extraction

A systematic search was conducted in PubMed, Embase, and the Cochrane Library in September 2025. The full search can be found at Supplementary Table 1. Data extraction was performed independently by two reviewers using a standardized template that included sample size, study design, participant characteristics, photobiomodulation parameters (wavelength, power, frequency, duration), control condition, outcome measures, and follow-up duration (W.A. and M.L.R.D.). Disagreements were resolved through authors consensus.

### Quality assessment

Risk of bias for included RCTs was independently assessed by two authors (K.S. and W.A.) using the Cochrane Risk of Bias Tool 2.0 (RoB 2). Items evaluated included randomization, allocation concealment, blinding of participants and personnel, outcome assessment, incomplete data, and selective reporting [55]. Any disagreements were resolved by discussion and consensus. Publication bias was not assessed with a funnel plot due to the final inclusion of fewer than 10 studies, in accordance with Cochrane recommendations [30].

### Statistical analysis

Meta-analyses were conducted using R software (version 4.3.2). For continuous outcomes, mean differences (MD) with 95% confidence intervals (CI) were calculated. Heterogeneity was assessed with Cochran’s Q test and quantified using the I^2^ statistic. A p-value > 0.10 and I^2^ < 25% were considered indicative of low heterogeneity. A random-effects model based on Restricted Maximum Likelihood (REML) estimation was applied. Leave-one-out sensitivity analyses were performed to evaluate the robustness of the findings.

## Results

### Study selection

**Fig. 1:**
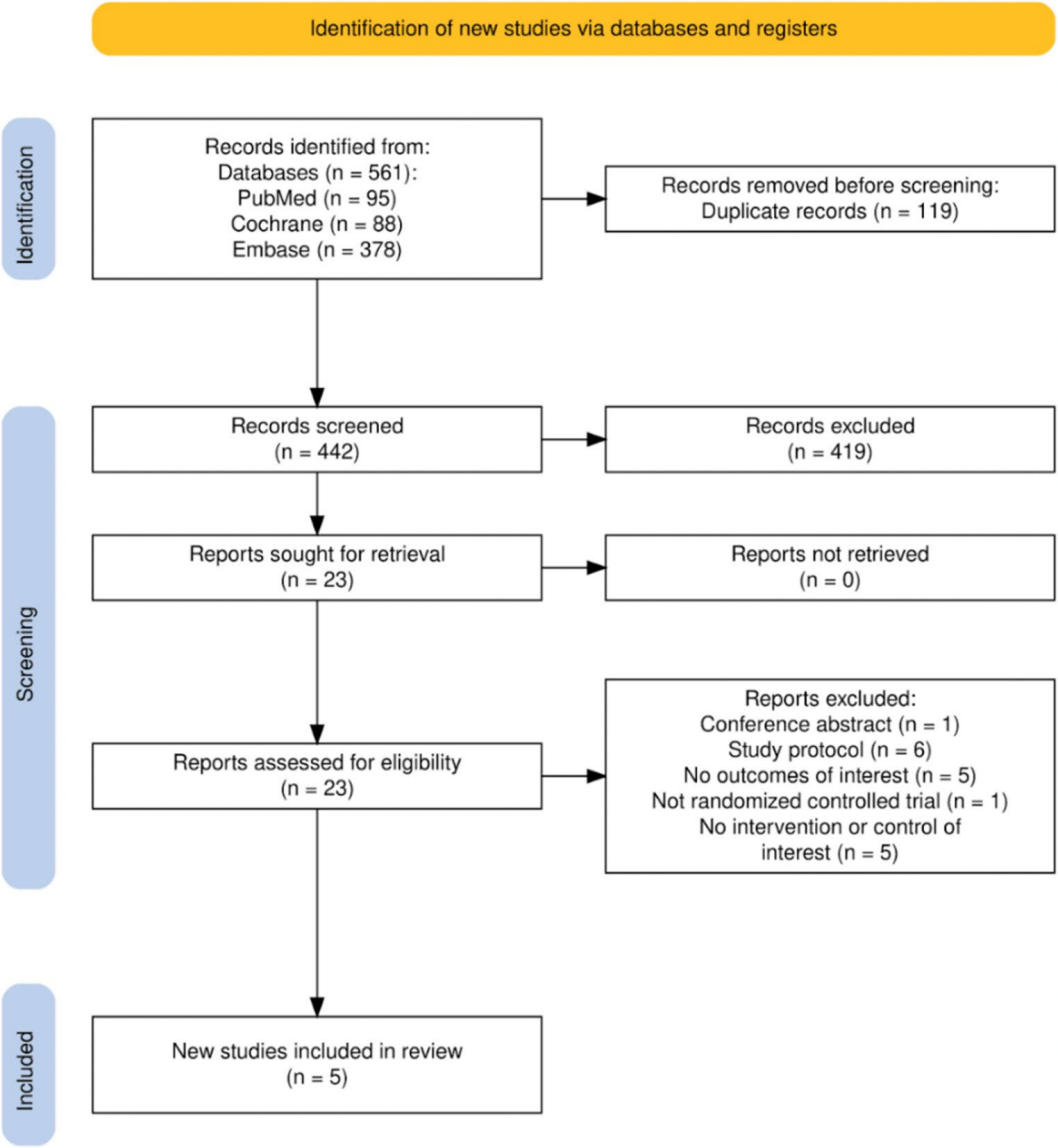
PRISMA flow diagram of study selection

The systematic search yielded 561 records across three major databases: PubMed (*n* = 95), Cochrane (*n* = 88), and Embase (*n* = 378). Following the removal of 119 duplicates, 442 unique records underwent title and abstract screening, from which 419 were excluded as irrelevant. The remaining 23 reports were retrieved in full and assessed for eligibility. Of these, 5 studies fulfilled all criteria and were included in the meta-analysis (Fig. 1).

### Study characteristics

Across the five trials, populations included patients with End-Stage Renal Disease (ESRD) on hemodialysis [11], chronic insomnia [12], xerostomia with hyposalivation [24], and major depressive disorder [28, 40]. Sample sizes ranged from 35 to 57 participants, with mean ages from 44.9 to 68.4 years and 65.5% female. Interventions encompassed multi-channel or diode lasers, headbands, and irradiation goggles, with wavelengths spanning 360–850 nm, and light outputs variously described by power density (W/cm^2^), energy density (J/cm^2^), or power (W) and energy (J) units. Session duration ranged from a few minutes to 12 h, while treatment periods lasted 4 to 12 weeks. Table 1 details the reported characteristics, number of participants in the intervention and control (I/C) groups, age, sex, intervention, laser parameters, session and treatment duration of the included studies.

**Table 1:** Characteristics of the included studies

Study	Population	Number of participants I/C	Mean age, years I/C	Female, % I/C	Intervention	Laser parameters	Session duration	Treatment duration
Chang et al., [11]	ESRD patients on hemodialysis	20/20	67.75/66.25	40/35	Three-channel laser device	830 nm 30 mW 10 Hz 20.55 mW/cm^2^	KI 1 and ST 36 acupoints: 30 min Palm: 10 min	4 weeks
Chen et al., [12]	Patients with chronic insomnia	23/12	50.99/52.17	83.3/86.9	12-beam Physiolaser Olympic system	600 mW 540 J	15 min 2 sessions per week	5 weeks
Ferrandez et al., [24]	Patients with xerostomia and hyposalivation	30/30	65.4/68.4	93.3/100	LaserSmile^®^ low power GaAlAs diode laser	810 nm 6 J/cm^2^	Parotid gland: 2 min and 24 s Submandibular gland: 1 min and 12 s 1 session per week	6 weeks (sleep outcomes reported at 2- and 6-week follow-ups)
Guu et al., [28]	Patients with MDD	25/23	48.48/49.35	44/61	Wearable self-administered headband	850 nm 40 Hz 18 mW/cm^2^	20 to 40 min 1 or 2 sessions daily	8 weeks
Noda et al., [40]*	Patients with MDD	57	44.9	47.4	Irradiation goggles	360–400 nm 310 µW/cm^2^ 40 Hz	3 h session daily	4 weeks

### Pooled analysis

**Fig. 2:**
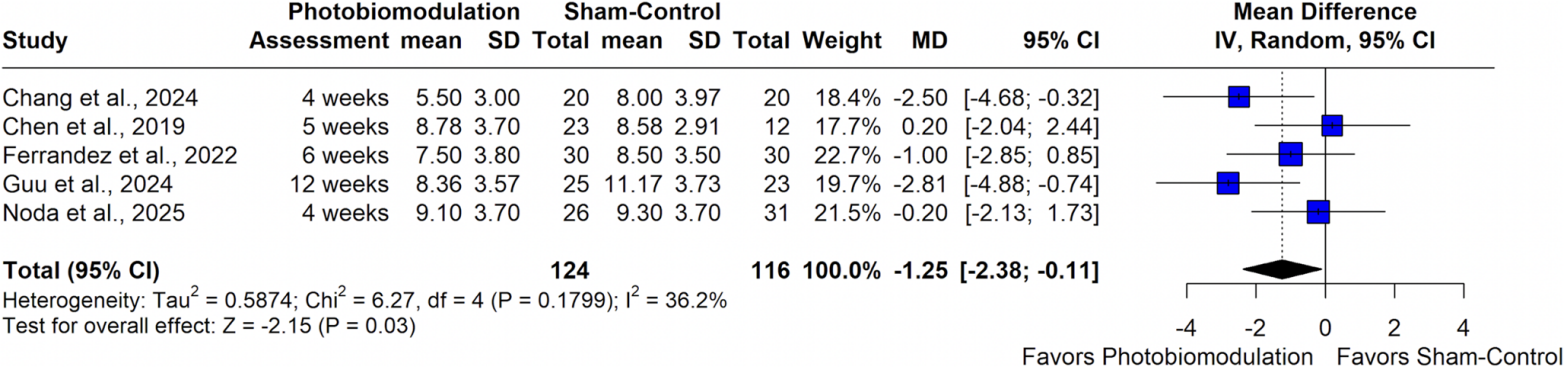
Forest plot showing the mean difference between photobiomodulation and sham control. The random-effects model favored photobiomodulation (MD = − 1.25; 95% CI: − 2.38 to − 0.11; *p* = 0.03) with moderate heterogeneity (I^2^ = 36.2%)

Five randomized controlled trials (total = 240 participants) compared photobiomodulation with sham-control in sleep quality. The pooled analysis showed photobiomodulation was associated with a statistically significant improvement in the Pittsburgh Sleep Quality Index (PSQI) compared with sham-control (MD = − 1.25; 95% CI − 2.38 to − 0.11; *p* = 0.03, I^2^ = 36.2%; Fig. 2). However, despite reaching statistical significance, the confidence interval was relatively wide (spanning 2.27 units on the mean difference of the score), indicating low precision for the overall estimate.

**Fig. 3:**
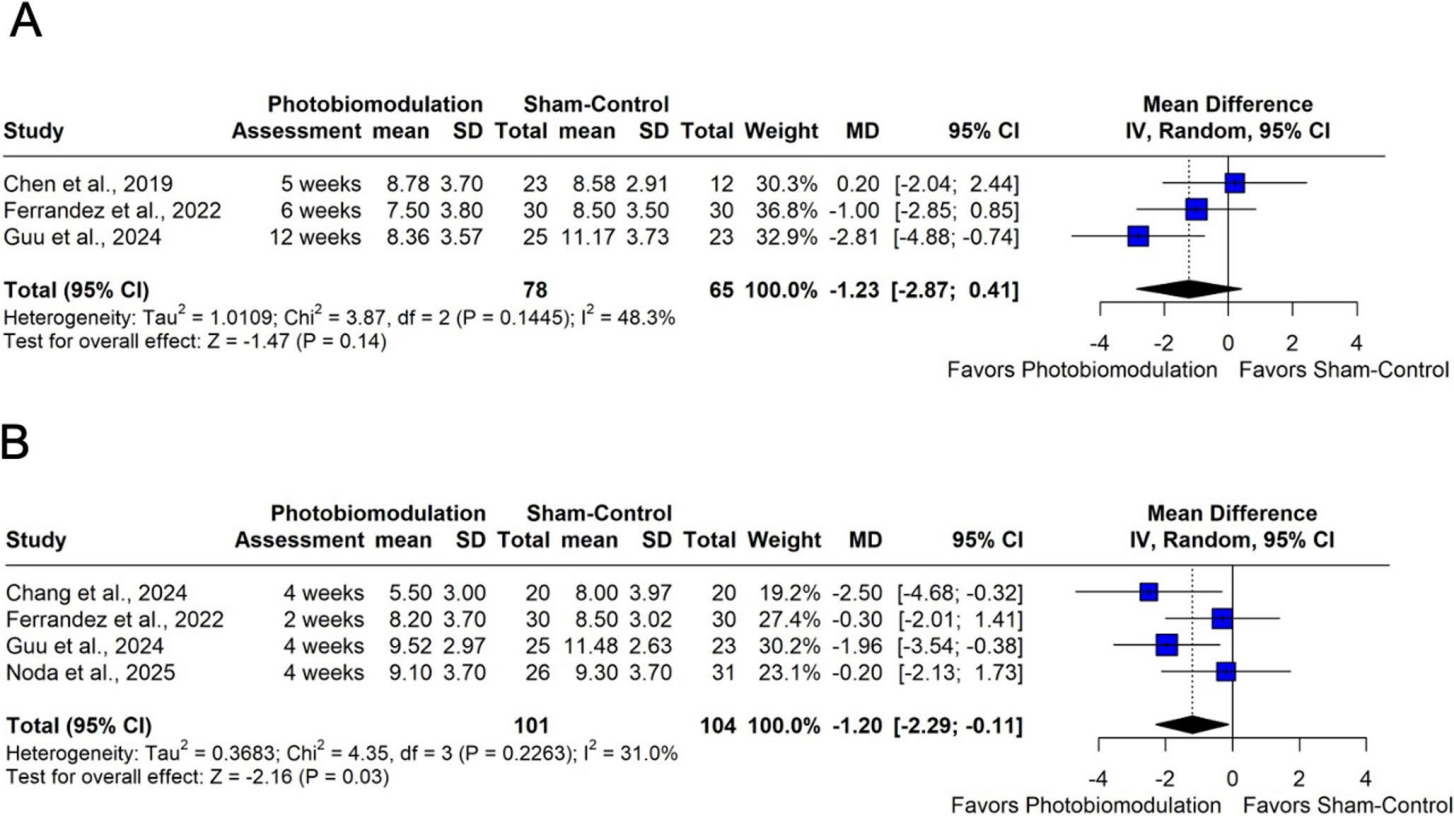
Subgroup meta-analysis comparing the effects of photobiomodulation and sham-control on sleep outcomes. **A** Studies with intervention durations ≥ 5 weeks showed no significant difference between groups (MD = − 1.23; 95% CI: − 2.87 to 0.41; *p* = 0.14; I^2^ = 48.3%). **B** Studies with shorter protocols (≤ 4 weeks) demonstrated a significant improvement in sleep quality favoring photobiomodulation (MD = − 1.20; 95% CI: − 2.29 to − 0.11; *p* = 0.03; I^2^ = 31%). These findings suggest that shorter photobiomodulation interventions may yield more consistent benefits across studies

Subgroup analyses were performed according to intervention duration. As shown in Fig. 3A, studies with photobiomodulation protocols lasting ≥ 5 weeks did not show significant differences compared to sham-control (MD = − 1.23; 95% CI: − 2.87 to 0.41; *p* = 0.14; I^2^ = 48.3%). In contrast, trials with shorter intervention durations (≤ 4 weeks) demonstrated a significant improvement in sleep quality favoring photobiomodulation (MD = − 1.20; 95% CI: − 2.29 to − 0.11; *p* = 0.03; I^2^ = 31%; Fig. 3B). The lower heterogeneity observed in this subgroup indicated that shorter duration photobiomodulation protocols showed more consistent and measurable benefits on sleep parameters across studies.

Leave-one-out sensitivity analysis confirmed the robustness of the findings, as exclusion of individual studies did not alter the direction of effect (Supplementary Fig. 1). The mean difference ranged from − 0.86 to − 1.55, consistently favoring photobiomodulation, with overlapping 95% CIs and low-to-moderate heterogeneity (I^2^ = 14.4%–51.5%). These results indicated that no single study disproportionately influenced the pooled estimate.

### Risk of bias assessment

All studies were considered at high risk of bias. The quality appraisal of each individual study is available in Supplementary Fig. 2.

## Discussion

In this systematic review and meta-analysis of five RCTs (*N* = 240), photobiomodulation significantly improved overall PSQI scores compared with sham-control. A subgroup analysis showed a modest, statistically significant benefit in trials with treatment periods of 4 weeks or less, whereas studies with treatment periods longer than 4 weeks demonstrated no significant effect. Although the included clinical trials did not directly investigate the neurobiological mechanisms underlying sleep improvement, evidence from preclinical and neuroimaging studies provides biologically plausible hypotheses that may help explain the observed findings [50, 62, 64].

Photobiomodulation is a non-invasive technique that primarily uses low-level red or near-infrared light to stimulate biological processes through mitochondrial mechanisms, particularly via photonic activation of cytochrome c oxidase [10, 61]. This mitochondrial stimulation enhances ATP production via oxidative phosphorylation, modulates reactive oxygen species (ROS), releases nitric oxide that promotes vasodilation and tissue oxygenation, and reduces neuroinflammation [10, 23, 62, 64]. These physiological effects may be relevant to the regulation of circadian rhythms and sleep architecture, particularly in populations with sleep disturbances due to chronic conditions or neuropsychiatric diseases [1, 3, 29]. Evidence from fibromyalgia research supports this rationale [15, 47]. For example, Ruaro et al. [47] demonstrated that photobiomodulation significantly reduced pain, fatigue, and sleep-related impairments, while da Silva et al. [15] reported synergistic effects of photobiomodulation combined with exercise training on pain thresholds, fatigue, and sleep quality. These findings reinforce that sleep benefits may emerge as part of a broader neuromodulatory effect on chronic multisystem conditions.

Neuroimaging and electrophysiological studies have further supported the potential of photobiomodulation to modulate neural networks involved in sleep regulation. Functional near-infrared spectroscopy and fMRI studies have demonstrated that photobiomodulation enhances prefrontal cortical activity and network efficiency, reflected by functional connectivity and improved neurocognitive performance in younger and older adults [50, 53, 59]. These regions are critical for maintaining sleep–wake stability and executive functions impaired in insomnia and mood disorders [32, 35, 39, 60]. Additionally, photobiomodulation-induced upregulation of mitochondrial activity in glial and neuronal populations has been hypothesized to contribute to the restoration of homeostatic sleep pressure through effects on astrocytic energy metabolism and adenosine signaling [17, 43, 54, 56]. Together, these preclinical and neuroimaging findings provide biologically plausible hypotheses suggesting that photobiomodulation could influence sleep regulation through cellular bioenergetic enhancement and large-scale neuromodulatory network reorganization. However, these mechanisms were not directly evaluated in the clinical trials included in this meta-analysis.

Despite these promising neurobiological mechanisms, the clinical outcomes across trials remain heterogeneous. Importantly, the included studies employed both transcranial and non-transcranial photobiomodulation approaches, which may have contributed to the clinical heterogeneity observed across trials. Variability in treatment parameters including wavelength, energy density, and cortical target may influence the magnitude and consistency of sleep-related effects. Studies employing prefrontal stimulation with higher fluence and shorter protocols (≤ 4 weeks) tended to report greater improvements in sleep quality, whereas longer or lower-intensity interventions often yielded null results [11, 24, 28, 40]. These findings highlight the need for standardized stimulation protocols and multimodal assessments integrating neuroimaging, electrophysiology, and behavioral outcomes to clarify the dose–response relationship between photobiomodulation and sleep regulation.

Although the current evidence suggests a possible role for photobiomodulation in sleep modulation, the findings should be interpreted with considerable caution. The small sample sizes, heterogeneity in intervention parameters, and limited blinding procedures across trials reduce the generalizability of findings. In addition, variability in sham-control designs across studies may have influenced participant blinding and expectancy effects, potentially affecting the perceived efficacy of active photobiomodulation interventions. The inclusion of both transcranial and non-transcranial photobiomodulation approaches, including laser acupuncture and peripheral irradiation protocols, may have introduced substantial clinical heterogeneity due to potentially distinct biological mechanisms underlying these interventions. The substantial variability in wavelengths across studies, ranging from violet/ultraviolet to near-infrared spectra, may also involve markedly different tissue penetration depths and biological targets, further contributing to mechanistic heterogeneity across interventions. All included studies were judged to have a high risk of bias, which further limits the reliability and interpretability of the pooled estimates. The search strategy employed in this review may also have been limited by the use of restricted search terms, which could have resulted in the omission of potentially relevant studies. The absence of objective sleep measures such as actigraphy or polysomnography in most studies restricts the ability to confirm physiological sleep improvements. The modest effect size observed in the pooled analysis and the wide confidence intervals also indicate limited precision of the current evidence. Although the pooled reduction in PSQI scores reached statistical significance, the observed mean difference (MD = − 1.25) may not reach the threshold generally considered clinically meaningful for the PSQI scale. Therefore, the present findings should not be overinterpreted as definitive evidence of clinical efficacy. Future studies should prioritize the use of standardized stimulation parameters, particularly regarding wavelength selection, irradiance, treatment duration, and cortical target regions. Based on the findings of the present review, shorter intervention protocols (≤ 4 weeks) and prefrontal stimulation approaches may represent promising targets for further investigation. Future trials should also clearly report irradiation parameters, including power density and energy dose, to improve reproducibility and allow more reliable comparisons across studies. Multimodal outcome measures combining subjective sleep questionnaires with objective assessments, such as polysomnography, actigraphy, electrophysiological recordings, and neuroimaging biomarkers, are also needed.

## Conclusion

In summary, this systematic review and meta-analysis provide preliminary and low-precision evidence suggesting that photobiomodulation may modestly influence subjective sleep quality outcomes, particularly in shorter-duration intervention protocols.

## Data Availability

All data used for the systematic review and meta-analysis are contained within the manuscript. 

## Supplementary Information

Supplementary Material 1. Table S1: Search strategy employed in the Cochrane Library, Embase, and PubMed databases.

Supplementary Material 2. Figure S1: Risk of bias assessment across the five Cochrane RoB 2.0 domains for the included randomized controlled trials. Domains: D1 – bias arising from the randomization process; D2 – bias due to deviations from intended intervention; D3 – bias due to missing outcome data; D4 – bias in measurement of the outcome; D5 – bias in selection of the reported result. Figure S2: Risk of bias assessment across the five Cochrane RoB 2.0 domains for the included randomized controlled trials. Domains: D1 – bias arising from the randomization process; D2 – bias due to deviations from intended intervention; D3 – bias due to missing outcome data; D4 – bias in measurement of the outcome; D5 – bias in selection of the reported result.

## Data Availability

All data used for the systematic review and meta-analysis are contained within the manuscript.
