# Supplementary material for: Photobiomodulation and sleep quality: systematic review and meta-analysis
Source: Lasers Med Sci. 2026 Jun 20;41(1):122. doi: 10.1007/s10103-026-04913-5 (PMC13283139; doi:10.1007/s10103-026-04913-5)
Supplement: Supplementary file 1 [file 10103_2026_4913_MOESM1_ESM.odt]

Supplementary Table 1. Search strategy employed in the Cochrane Library, Embase, and PubMed databases. 

| Cochrane Embase PubMed | (photobiomodulation OR "low level laser therapy") AND sleep |
| --- | --- |

Legend: The Boolean combination used was (photobiomodulation OR "low level laser therapy") AND sleep, adapted to the syntax of each database. Searches were limited to human and animal studies, with no language or date restrictions.
