# Supplementary material for: Photobiomodulation and sleep quality: systematic review and meta-analysis
Source: Lasers Med Sci. 2026 Jun 20;41(1):122. doi: 10.1007/s10103-026-04913-5 (PMC13283139; doi:10.1007/s10103-026-04913-5)
Supplement: Supplementary file 2 [file 10103_2026_4913_MOESM2_ESM.odt]

Supplementary Figure 1. Sensitivity analysis assessing the influence of each study on the overall effect size. Sequential omission of individual trials did not materially alter the pooled estimate (MD range: –0.86 to –1.55), confirming the robustness of the results. Heterogeneity values (I2 = 14.5–51.5%) and p-values remained stable across iterations, indicating no single study had a disproportionate impact on the overall meta-analytic model.

Supplementary Figure 2. Risk of bias assessment across the five Cochrane RoB 2.0 domains for the included randomized controlled trials. Domains: D1 – bias arising from the randomization process; D2 – bias due to deviations from intended intervention; D3 – bias due to missing outcome data; D4 – bias in measurement of the outcome; D5 – bias in selection of the reported result.
